# Estradiol and progesterone affect enzymes but not glucose consumption in a mink uterine cell line (GMMe)

**DOI:** 10.1042/BSR20193512

**Published:** 2020-04-24

**Authors:** Hayden Holmlund, Álvaro Marín-Hernández, Jennifer R. Chase

**Affiliations:** 1 Northwest Nazarene University, 623 S. University Blvd, Nampa, ID 83686, U.S.A.; 2 Departamento de Bioquímica, Instituto Nacional de Cardiología, Mexico City 14080, México

**Keywords:** Enzyme kinetics, estradiol, glycolysis, progesterone

## Abstract

Cells lining the uterus are responsible for storage and secretion of carbohydrates to support early embryonic development. Histotrophic secretions contain glycogen and glycolytic products such as lactate and pyruvate. Insufficient carbohydrate storage as glycogen has been correlated with infertility in women. While it is clear that changes in estrogen (17-β-estradiol (E_2_)) and progesterone (P_4_) *in vivo* affect the distribution of glucose in the uterine cells and secretions, the biochemical mechanism(s) by which they affect this crucial allocation is not well understood. Furthermore, in cultured uterine cells, neither E_2_ nor P_4_ affect glycogen storage without insulin present. We hypothesized that P_4_ and E_2_ alone affect the activity of glycolytic enzymes, glucose and glycolytic flux to increase glycogen storage (E_2_) and catabolism (P_4_) and increase pyruvate and lactate levels in culture. We measured the rate of glucose uptake and glycolysis in a mink immortalized epithelial cell line (GMMe) after 24-h exposure to 10 μM P_4_ and 10 nM E_2_ (pharmacologic levels) at 5 mM glucose and determined the kinetic parameters (*V*_max_, *K*_m_) of all enzymes. While the activities of many glycolytic enzymes in GMMe cells were shown to be decreased by E_2_ treatment, in contrast, glucose uptake, glycolytic flux and metabolites levels were not affected by the treatments. The cellular rationale for P_4_- and E_2_-induced decreases in the activity of enzymes may be to prime the system for other regulators such as insulin. *In vivo*, E_2_ and P_4_ may be necessary but not sufficient signals for uterine cycle carbohydrate allocation.

## Introduction

A fertilized human embryo is estimated to have only a 30% chance of implanting in the uterine wall [1]. Failure of implantation is approximately 30–40% for fertilized embryos in cattle, with a lower rate estimated for sheep, which is highly correlated to circulating progesterone (P_4_) [2]. It is likely that some of these losses are the result of inadequate P_4_ and 17-β-estradiol (E_2_)-stimulated nutrient storage by uterine glands and/or failure to mobilize the nutrients toward the uterine lumen as the embryo is rapidly growing until it implants in the uterine wall. Before implantation, lactate and pyruvate are essential for development of mammalian embryos to *at least* the morula stage [3,4]. It is thus essential to assess the cellular mechanisms that regulate the production of these molecules in order to understand how to maximize fertility.

Glandular epithelial cells are responsible for supplying nutrients to the embryo during the first trimester of pregnancy in a process that is coordinated by both P_4_ and E_2_. In humans, P_4_ produced by the corpus luteum promotes the secretory transformation of the endometrial epithelial glands that will, in response to E_2_, produce a uterine milk called histotroph containing glucose, lactate, pyruvate and amino acids which support the developing embryo at least until placentation is complete [5,6]. In mustelids, E_2_ levels peak before ovulation and rise with P_4_ levels, which are highest during peri-implantation [7].

P_4_ and E_2_ affect glucose metabolism during the uterine cycle *in vivo*. E_2_ promotes glycogen storage in mammals *in vivo*, while elevated P_4_ promotes catabolism of the stored carbohydrate [8–11]. Glycolysis, too, is highest in estrus, when E_2_ is elevated in bovine [12] and rat uterus [13]. In rats, all glycolytic enzymes increase their activity in response to estrogen [14], while P_4_ counters the stimulatory effects of E_2_ on pyruvate kinase (PYK; E.C. 2.7.1.40) and hexokinase (HK; E.C. 2.7.1.1) activities [15,16]. However, in sheep the activities of lactate dehydrogenase (LDH, E.C. 1.1.1.27) and glucose-6-phosphate dehydrogenase (G6PDH; E.C. 1.1.1.49) did not change significantly during the estrus cycle [17,18]. Enzyme activities have not been reported for specific endometrial cell types under physiological conditions and kinetic parameters have not been thoroughly studied in endometrial tissue of any organism.

The American mink (*Neovison vison*) is a significant model organism in the study of uterine carbohydrate metabolism in reproduction. Mink exhibit an obligatory embryonic diapause, often having blastocysts suspended 50–60 days *post-coitum.* P_4_ stimulates uterine glycogen catabolism in the mink endometrium, whereas E_2_ promotes glycogen accumulation *in vivo* [19,20] and in cultured cells in the presence of insulin. The mechanisms by which E_2_ and P_4_ directly affect glycolysis and its enzymes in uteri have not been determined in mink and other mammals because of the confounding effect of hormones such as insulin.

Mink immortalized endometrial cells (GMMe) [21] provide an ideal model system to study hormonal regulation of glycolysis in the uterus, not only because carbohydrate metabolism has already been studied in mink uterus and in GMMe, but also because they are one of the only immortalized endometrial cell lines that are commercially available. They respond to E_2_ and P_4_ [22,23] and express estrogen receptor Type 1 [22]. They also represent a source of mink enzymes, for which few *K*_m_s have been determined under any conditions [24]. This also isolates the behavior of epithelial cells where most of the glycogen is stored [19,20], compared with the *in vivo* studies which work with homogenized uterine tissue containing both stromal and epithelial tissues.

P_4_ and E_2_ affect enzymes involved in glucose and glycogen metabolism in the mink endometrium. Glycogen phosphorylase and HK-1 levels are highest during estrus and diapause, glucose-6-phosphatase levels are highest during diapause, while glycogen synthase levels are undetectable after estrus [20]. However, hormonal regulation has not been studied for mink glycolytic enzymes other than HK and no kinetic studies have been done in GMMe. Measuring glycolytic enzyme kinetics will help determine *how* P_4_ and E_2_ affect carbohydrate allocation in mink uterine cells.

Though P_4_ and E_2_ are essential for changing carbohydrate usage and enzymes in mink endometrial tissue, *in vitro* it has been shown that E_2_, at least, must act in coordination with other hormones (primarily insulin) to affect glycogen levels. In GMMe, E_2_ enhances insulin’s stimulatory effect on glycogen storage, while E_2_ alone has no effect on glycogen levels [22]. Thus, any changes to glycolytic enzymes by P_4_ and E_2_ alone may not affect carbohydrate usage directly, but rather play a larger role in priming the system for hormonal regulation by insulin.

The enzyme mRNA level is often used as a proxy for levels of enzymes, but a quantitative estimation of their maximum velocities requires direct measurement of their activity. This is the first study of glycolysis and glycolytic enzyme kinetics in immortalized endometrial cells. We measured the rate of glycolysis in GMMe cells after 24 h of exposure to 10 μM P_4_ and 10 nM E_2_ at 5 mM glucose and the kineticparameters (*V*_max_, *K*_m_) of glycolytic enzymes, phosphoglucomutase (PGM; E.C. 5.4.2.2) and G6PDH spectrophotometrically with coupled assays. The activity of several glycolytic enzyme activities in GMMe cells were decreased by E_2_ treatment, whereas their *K*_m_s were increased by P_4_ treatment though glucose uptake, glycolytic flux and metabolite values were unchanged by the treatments.

## Methods

NADP^+^ and NAD^+^ were purchased from Research Products International. All other chemicals and enzymes were purchased from Sigma–Aldrich. The concentrations in stock solutions of substrates were calibrated spectrophotometrically by enzymatic assays, monitoring changes in NAD(P)H using ε = 6.22 mM^−1^.cm^−1^.

### Cell culture

GMMe (ATCC® CRL-2674™) cells were used between passages 4 and 19 and cultured in growth media (DMEM/F-12, 5% FBS, 1% pen/strep, 16 mM glucose, no Phenol Red; #DFL-14, Caisson Labs, Smithfield, UT) in humidified air: 95%, CO_2_: 5%, 37°C in 75- or 175-cm^3^ culture flasks. Medium was refreshed every ∼3 days and replaced with sterile medium. Cells were passaged when ∼90% confluent at a subcultivation ratio of 1:2 to 1:6. For the last 24 h, the medium was replaced with DMEM/F-12 (5 mM glucose) every 12 h, with 10 nM E_2_, 10 μM P_4_ or dimethyl sulfoxide (DMSO; control) vehicle. These hormone levels were shown to be the minimum necessary to affect insulin receptor and, with insulin, glycogen levels in GMMe cells [22,23]. These levels were several orders of magnitude smaller than what was measured *in vivo* in mink [25]. E2 (E2758, Sigma–Aldrich) and P_4_ (P783; Sigma–Aldrich) were dissolved in 100% DMSO (Sigma–Aldrich, Hybri-Max™) to make stock solutions (50 μM E_2_ and 100 mM P_4_). Medium glucose at 5 mM was used [22], which is similar to blood glucose in mink during the breeding season (∼5.3 mM) [26,27].

Before harvesting, the cell layer was washed with 3–6 ml of PBS to remove all media. The cells were incubated with 0.25% (w/v) trypsin and 0.53 mM EDTA solution (3–6 ml) for <10 min to dislodge. The cells from this medium, along with one wash of the flask with 5 ml PBS, were pelleted at 2000×***g*** for 5 min, resuspended in PBS to wash and again pelleted/resuspended one to two times. The supernatant was removed, and the cells were resuspended in Krebs–Ringer. This cell suspension was used for determination of maximum velocity in total cells. Harvested cell suspensions were assayed for total protein content using a bicinchoninic acid (BCA) assay [28] and for cell number using a cell counter (Countess II, Life Technologies, Thermo Fisher).

To prepare cytosol-enriched fraction for determination of *K*_m_ values, cells were resuspended in enzyme extract buffer (1 ml of 25 mM Tris/HCl, pH 7.6, plus 5 mM DTT, 1 mM EDTA and 1 mM PMSF). The cellular suspension was frozen in liquid N_2_, then thawed at 37°C for four cycles. The cellular lysates were centrifuged at 14000 rpm for 20 min and 4°C. Afterward, the supernatant (i.e., the cytosolic-enriched fractions) was collected and mixed with glycerol (10%, v/v, final concentration). The protein content was determined by the BCA assay. The material was aliquoted (<1 ml) and stored at −70°C until use.

### Glucose uptake

Glucose uptake by GMMe cells was measured using UptakeGlo Assay (Promega, U.S.A.). Briefly, GMMe cells were seeded (20000 cells/well) in triplicate for each independent cell batch assayed, in a 96-well plate and provided high-glucose medium. After 24 h, the medium was replaced with treatment medium (DMEM/F12, 5 mM glucose, with 10 nM E_2_, 10 μM P_4_, or DMSO vehicle) which was replaced after 12 h. PBS-washed cells were incubated for 10 min in glucose-deprived medium supplemented with 1 mmol/l 2-Deoxy-d-Glucose. Afterward, cells were lysed and the accumulation of 2-deoxy-glucose 6-phosphate (G6P) was detected using G6PDH and luciferase, which uses the produced NADPH as a substrate. Glucose uptake (nmol/mg/min) was calculated from the time of uptake, the average protein content of matched wells and the amount of 2-deoxy-G6P accumulated. A standard curve of 2DG6P was used to calculate the amount of 2-deoxy-G6P accumulated.

### Enzyme assays

Glycolytic enzyme activities and affinity constants were determined for the forward (glycolytic) and reverse (gluconeogenic) reactions. Kinetic parameters (*V*_max_, *K*_m_) were determined at 37°C following the NAD(P)^+^ reduction or nicotinamide adenine dinucleotide phosphate (NADP(H)) oxidation at 340 nm in a HP8453 spectrophotometer (Agilent, Santa Clara, CA) with an eight-cell carrier. All reactions were started by adding the specific substrates. When the specific substrate was omitted from the assay mixture, enzyme activity was absent. In all assays, control experiments were carried out to establish the linearity range of enzyme activities regarding protein added. Initial rates were calculated only the linear part of activity curve using ChemStation software (Agilent, Santa Clara, CA). All assays were carried out in KME buffer (100 mM KCl, 50 mM MOPS, 0.5 mM EGTA, pH 7.0) with the exception of LDH assay in reverse reaction, for which the assay buffer contained 0.4 M hydrazine/0.5 M glycine, pH 9.

*V*_max_ values were measured in cell suspension by lysing the cells in 0.02% Triton X-100 to obtain the corresponding *V_max_* total cellular protein units and know the amount of total active enzyme present in the cells. In order to correctly estimate the *V_max_*, the reactions were carried out at saturated concentrations of substrates (at least ten-times *K*_m_). All enzyme activities were measured within 3 h after the cells were harvested.

The *K*_m_ values were determined in cytosol-enriched fractions by varying the substrate concentrations, as is indicated for each enzyme assay, to ensure that they varied from <*K*_m_ to approximately saturation for each enzyme.

The HK activity assay was carried out in the presence of 10 U G6PDH, 500 µM NADP^+^, 15 mM MgCl_2_, 150–300 µg of cell protein and variable adenosine triphosphate (ATP; 0.2–10 mM at constant 3 mM glucose) or glucose (0.02–3 mM at constant 10 mM ATP) to determine *K_m_* values for ATP and glucose, respectively; the reaction was started by adding glucose. When HK *V_max_* was determined, the ATP and glucose concentrations were 10 and 3 mM, respectively. The reaction was linear for approximately 500–2000 s.

The PGM activity assay for the reverse reaction (glucose 1-phosphate (G1P) → G6P) was carried out in the presence of 10 U G6PDH, 500 µM NADP^+^, 2 mM MgCl_2_, 2 µM G1, 6BP and 150–300 µg of cell protein; the reaction was started by adding G1P (0.01–10 mM) to determine *K*_m_ value for G1P. When PGM *V_max_* was determined, the G1P concentration was 10 mM. The reaction was linear for approximately 500–2000 s.

The G6PDH activity assay was carried out in the presence of 150–300 µg of cell protein, and variable NADP^+^ (10–700 µM at constant 400 µM G6P) or G6P (1–400 µM at constant 700 µM NADP^+^) to determine *K*_m_ values for NADP^+^ and G6P, respectively; the reaction was started by adding G6P. When G6PDH *V_max_* was determined, the NADP^+^ and G6P concentrations were 500 and 100 µM, respectively. The reaction was linear for approximately 500–2000 s.

The phosphohexose isomerase (HPI; E.C. 5.3.1.9) activity assay for reverse reaction (fructose 6-phosphate (F6P) → G6P) included 10 U G6PDH, 500 µM NADP^+^ and 15–30 µg of cell protein; the reaction was started by adding F6P (0.01–2 mM) to determine *K*_m_ value for F6P. When HPI *V_max_* was determined, the F6P concentration was 2 mM. The reaction was linear for approximately 50–500 s.

The phosphofructokinase type I (PFK-1; E.C. 2.7.1.11) *V_max_* assay used 10 U aldolase (ALDO; E.C. 4.1.2.13), 10 U α-glycerophosphate dehydrogenase, 10 U triosephosphate isomerase (TIM; E.C. 5.3.1.1), 5 mM MgCl_2_, 150 µM NADH, 150–300 µg of cell protein and ATP (700 µM at constant 20 mM F6P); the reaction was started by adding F6P. The F6P and ATP concentrations were 20 mM and 700 µM, respectively. The reaction was linear for approximately 500–2000 s.

The ALDO activity assay used 10 U α-glycerophosphate dehydrogenase, 10 U TIM, 150 µM NADH and 60–120 µg of cell protein; the reaction was started by adding fructose 1,6-bisphosphate (F1,6BP; 0.5–300 µM) to determine *K_m_* value for F1,6BP. When ALDO *V*_max_ was determined, the F1,6BP concentration was 300 µM. The reaction was linear for approximately 500–2000 s.

The TIM activity assay for forward reaction (TIM*f*, dihydroxyacetone phosphate (DHAP) → glyceraldehyde 3-phosphate (G3P)) used 10 U of glyceraldehyde-3-phosphate dehydrogenase (GAPDH), 1 mM NAD^+^, 10 mM arsenate (AsO_4_), 5 mM cysteine and 15–30 µg of cell protein; the reaction was started by adding DHAP (0.5–30 mM) to determine *K*_m_ value for DHAP. When TIM *V*_max_ forward was determined, the DHAP concentration was 30 mM. The reaction was linear for approximately 50–500 s.

The TIM activity assay for reverse reaction (TIM*r*, G3P → DHAP) used 150 µM NADH, 2.5 mM EDTA, 10 U α-glycerophosphate dehydrogenase and 5–10 µg of cell protein; the reaction was started by adding G3P (0.1–4.5 mM) to determine *K_m_* value for G3P. The reaction was linear for approximately 50–500 s.

The phosphoglycerate kinase (PGK; E.C. 5.4.2.1) activity assay for reverse reaction (PGKr, 3-phosphoglycerate (3PG) → 1,3-bisphosphoglycerate (1,3BPG)) used 10 U GAPDH, 10 mM ATP, 15 mM MgCl_2_, 150 µM NADH and 150–300 µg of cell protein; the reaction was started by adding 3PG (0.5–10 mM) to determine *K_m_* value for 3PG. When PGK *V*_max_ reverse was determined, the 3PG concentration was 10 mM. The reaction was linear for approximately 500–2000 s.

The phosphoglycerate mutase (PGaM; E.C. 2.7.2.3) activity assay used 10 U enolase (ENO; E.C. 4.2.1.11, 10 U PYK, 10 U LDH, 1 mM adenosine diphosphate (ADP), 5 mM MgCl_2_, 150 µM NADH and 150–300 µg of cell protein; the reaction was started by adding 3PG (0.001–3 mM) to determine *K*_m_ value for 3PG. When PGaM *V*_max_ was determined, the 3PG concentration was 3 mM. The reaction was linear for approximately 500–2000 s.

The ENO activity assay was performed using 10 U PYK, 10 U LDH, 1 mM ADP, 5 mM MgCl_2_, 150 µM NADH and 60–120 µg of cell protein; the reaction was started by adding 2-phosphoglycerate (2PG; 1–200 µM) to determine *K*_m_ value for 2PG. When ENO *V*_max_ was determined, the 2PG concentration was 200 µM. The reaction was linear for approximately 500–2000 s.

The PYK activity assay used 10 U LDH, 5 mM MgCl_2_, 150 µM NADH, 7.5–15 µg of cell protein and variable ADP (0.1–1 mM at constant 600 µM PEP) or PEP (10–600 µM at constant 1 mM ADP) to determine *K*_m_ values for ADP and PEP; the reaction was started by adding PEP. When PYK *V*_max_ was determined, the ADP and PEP concentrations were 1 mM and 600 µM, respectively. The reaction was linear for approximately 50–500 s.

The LDH activity assay for forward reaction (LDH*f*, pyruvate → lactate) used 7.5–15 µg of cell protein and variable NADH (0.5–150 µM at constant 300 µM pyruvate) or pyruvate (10–300 µM at constant 150 µM NADH) to determine *K*_m_ values for NADH and pyruvate; the reaction was started by adding pyruvate. When LDH *V*_max_ forward was determined, the NADH and pyruvate concentrations were 150 and 300 µM, respectively. The reaction was linear for approximately 50–500 s.

The LDH activity assay for reverse reaction (LDH*r*, lactate → pyruvate) was performed with 15–30 µg of cell protein and variable NAD^+^ (10–500 µM at constant 30 mM lactate) or lactate (0.5–30 mM at constant 150 µM NAD^+^) to determine *K*_m_ values for NAD^+^ and lactate; the reaction was started by adding lactate. When LDH *V_max_* reverse was determined, the NAD^+^ and lactate concentrations were 500 µM and 30 mM, respectively. The reaction was linear for approximately 50–500 s.

Kinetic curves were fitted to the Michaelis–Menten equation using SigmaPlot 12.0 (Systat, San Jose, CA). Lineweaver–Burk plots were used to determine how many isoforms of HK, PGM, ALDO, ENO and PYK were present in GMMe cells.

### Glycolytic flux

Glycolytic flux was measured in two ways, by fluorescent measurement of acidification and by quantifying the production of lactate. The pH-Xtra Glycolysis Assay kit (#PH-200-4, Agilent, Santa Clara) was used to determine acidification rate. Briefly, GMMe cells from three distinct cell batches were seeded in triplicate in 96-well plates (100000 cells/well) and grown overnight in high-glucose medium. After 24 h, the medium was replaced with treatment medium (in triplicate, with 10 nM E_2_, 10 μM P_4_, or DMSO vehicle) which was replaced after 12 h. Each well was washed twice in provided respiration buffer then 90 μl of respiration buffer was added to each well (100 μl to control wells). To each experimental well, 10 μl of ‘pH-xtra reagent’ (fluorescent probe) was added. The plate was read immediately at 37°C in a BioTek H1M plate reader and the change in fluorescent lifetime from 100 to 300 µs delay and a 30-µs read window (excitation 360 nm, emission 620 nm). The emission lifetime (μs/h) values were corrected based on the signal control wells then scaled to [H^+^] by Agilent’s ‘Data Visualization tool’. The protons produced per hour per milligram of protein was calculated using the protein concentration (BCA assay) in matched wells.

For direct quantification of lactate production, cells (∼1 mg/ml in Krebs–Ringer buffer) were shaken in a water incubator (150 rpm) at 37°C in glass scintillation vials. After 10 min, a 100-μl sample was removed and vortexed with ice-cold PCA/EDTA (final: 3% PCA/1 mM EDTA). Glucose was added to a final concentration of 5 mM and the cells shaken for an additional 20 min. Cells (900 μl) were immediately mixed with PCA/EDTA (final concentration 3% PCA/1 mM EDTA) to stop all metabolism [29].

Universal pH indicator (Fisher) was added (10–25 μl) to each sample. Samples were neutralized with 3 M KOH/0.1 M Tris and left on ice for 45 min to overnight. Protein precipitate was pelleted at 14000 rpm for 40 min. The supernatant was stored at −20°C until it was assayed [29].

Total lactate before and after the 20 min was determined by standard coupled assays with 25 U LDH (from Rabbit muscle, Roche) in 0.4 M hydrazine/0.5 M glycine, pH 9 buffer with 1 mM NAD^+^ as substrate. The net change in absorbance at 340 nm was measured in a BioTek Synergy H1M in 96-well polystyrene plates with the pathlength-corrected for each well in duplicate. The rate of lactate produced was calculated as the difference between the initial and final samples, standardized to the amount of protein per minute. The glycolytic flux was calculated for each condition as the geometric mean and standard deviation of the log of the distribution for three independent cell batches assayed.

Steady-state phosphoenolpyruvate (PEP) and pyruvate concentrations were measured in the same supernatant using coupled assays in HEPES buffer (50 mM HEPES, 1 mM EGTA, pH 7.4) with 0.45 mM NADH, 2.5 mM inorganic phosphate (Pi), 1 mM ADP and 10 mM MgCl_2_. The reaction was started by adding 10 U PYK that quantified the amount of PEP and after LDH (2.5 U) that consumed the pyruvate. The concentrations were determined and changes in absorbance at 340 nm were measured, in duplicate, in a BioTek Synergy H1M in 96-well polystyrene plates with the pathlength corrected for each well. The intracellular concentrations were calculated from the amount of protein (considering that 2.28 μl/1.8 mg of cellular protein [30]).

### Statistical analysis

*t* tests were used for non-paired samples, with <0.05 being the criterion for significance. For metabolites and fluxes, *t* tests used the log values.

## Results

### Enzyme kinetics

**Figure 1:**
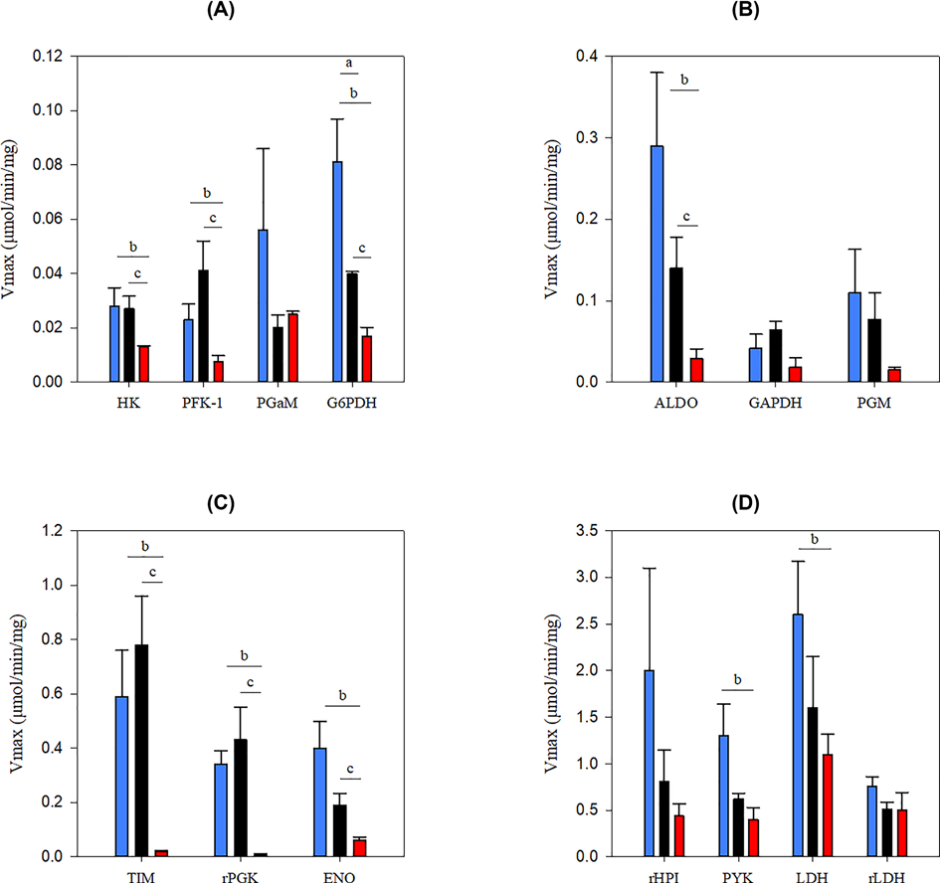
Maximum velocities of glycolytic enzymes Average maximum velocities shown (±SEM of three independent preparations assayed) with 2% Triton X-100. (a vs. control, b vs. E_2_, c P_4_ vs E_2_, *P*<0.05). Control (blue), P_4_ (black), E_2_ (red). **(A–D**) grouped by increasing *V*_max_.

E_2_ treatment significantly decreased (17–60%) the maximum velocities of HK, G6PDH, PFK-1, ALDO, TIM, PGK and ENO relative to both P_4_ treatment and control (Figure 1A–C). Additionally, the activities of PYK and LDH were significantly lower (27–42%) with E_2_ treatment than control (Figure 1D). Only G6PDH was lower (50%) with P_4_ treatment than control (Figure 1A). For all conditions, HK and PFK-1 had, on an average, the lowest maximum velocities, although exposure to E_2_ did lower the maximum velocities of TIM and PGK to similar levels (<20 nmol/min/mg cell protein) (Figure 1A,C). Such decreases in glycolytic enzyme levels might modify the concentrations of glycolytic metabolites and glycolytic flux, if these enzymes exert control on glycolytic flux.

The Michaelis constants (*K_m_s*) were also measured to provide a complete kinetic data set for mammalian glycolysis (Supplementary Figure S1A–M). P_4_ treatment significantly increased (2–5.5 times) the *K_m_s* of PGM, ENO and LDH (*Km*_G1P_, *Km*_2PG_ and *Km*_Pyr_), but decreased (14–48%) the *K_m_s* of G6PDH (*Km*_G6P_ and *Km*_NADP_) in comparison with control (Table 1). E_2_ treatment significantly increased (1.4–46 times) *Km*_G1P_, *Km*_2PG_ and *Km*_Pyr_ (Table 1). For E_2_ treatment, *Km*_2PG_ and *Km*_Pyr_ were significantly lower (37–66%) than P_4_ treatment, while *Km*_G1P_ was 23-times higher (Table 1).

**Table 1:** *K*_m_ values determined in cytosol-enriched fraction from GMMe cells (±SD) and from the BRENDA enzyme database (www.brenda-enzyme.org) for *Rattus norvegicus*

Enzyme		Control	10 µM P_4_	10 nM E_2_	*R. norvegicus* range
HK	*Km_gluc_*	91 ± 17	110 ± 42	63 ± 15	25–150
	*Km_ATP_*	830 ± 250	540 ± 180	ND	400–700
G6PDH	*Km_G6P_*	45.5 ± 0.6	22 ± 9.0^1^	37 ± 19	2–70
	*Km_NADP_*	40 ± 15	5.8 ± 3.3^1,2^	68 ± 37 (4)	0.3–23
PGM	*Km_G1P_*	26 ± 15	52 ± 9.0^1^	1200 ± 350^2,3^	Na
HPI	*Km_F6P_*	61 ± 18	200 ± 22 (2)^1,2^	88 ± 43	Na
ALDO	*Km_F1,6bP_*	11 ± 1.7	16 ± 15	ND	8.9
TIM	*Km_G3P_*	380 ± 290	ND	ND	Na
	*Km_DHAP_*	2200 ± 1800	ND	ND	Na
PGK	*Km_3PG_*	1700 ± 510	ND	ND	1400–1700
PGaM	*Km_3PG_*	5.9 (1)	20 (1)	ND	Na
ENO	*Km_2PG_*	4.9 ± 1.2	27 ± 6.7^1^	11 ± 2.1^2,3^	36–140
PYK	*Km_PEP_*	230 ± 19	210 ± 290	ND	180–960
	*Km_ADP_*	230 ± 110	260 ± 120	ND	80–1500
LDH	*Km_Pyr_*	65(2)	140 ± 18^1^	92 ± 27^3^	Na
	*Km_NADH_*	3.4 (2)	15 ± 17	ND	Na
	*Km_Lac_*	6500 ± 1200	5700 ± 3200	ND	1800
	*Km_NAD_*	170 ± 39	89 ± 50	ND	Na

For all GMMe parameters, *n*=3 unless noted in parentheses (1 vs. control, 2 vs. E_2_, 3 P_4_ vs E_2_, *P*<0.05). *n* is the number of independent cell batches assayed. *K*_m_ values in µM*.* Abbreviations: Na, no data in BRENDA database; ND, not determined.

Multiple isoforms have been identified in mammalian uterine tissue for PGM, ALDO, PYK, ENO and HK. We did not observe more than one isoform for each of these enzymes in any condition because there was no significant change in the slope of the Lineweaver–Burk plots of any enzyme (r^2^ ≥ 0.8) (Figure 3, Supplementary Figure S2). Although only one isoform of each enzyme was detected, the changes observed in the kinetic parameters might be altered by post-translational modifications (phosphorylation or acetylation), modifying enzyme expression levels or by completely changing which isoform is expressed.

### Glucose uptake and glycolytic flux

**Figure 2:**
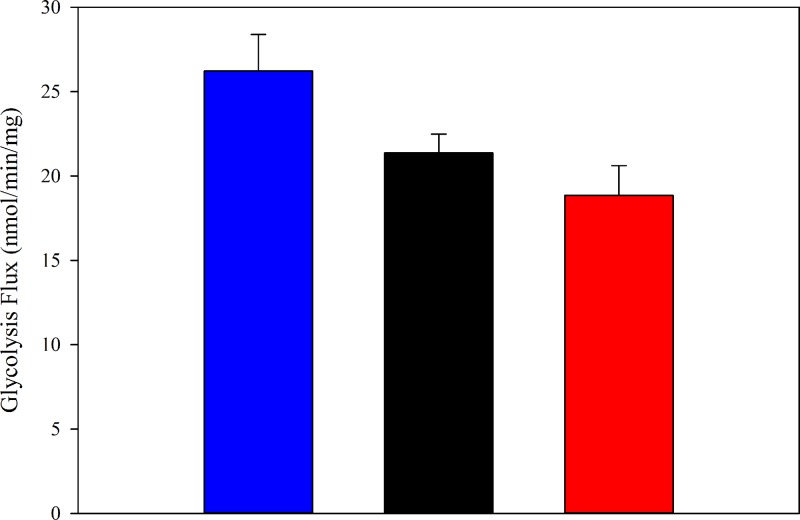
Glycolytic flux Geometric mean of lactate production in 20 min at 5 mM glucose (±SD, *n*=4 for control and P_4_; *n*=5 for E_2_). Control (blue), P_4_ (black) and E_2_ (red). *n* is the number of independent cell batches assayed.

Fluorescent determination of relative proton production from glycolysis showed no difference between the treatments with vehicle, P_4_ or E_2_ (7.3 ± 0.9, 6.6 ± 1 and 6.2 ± 1.4 pmol/min/mg protein, respectively). Lactate concentration increased linearly over at least 20 min, consistent with the system being in steady state. For control, the lactate levels in GMMe during the 20-min incubation in 5 mM glucose increased from 0.32 to 0.63 mM. The glycolytic flux did not significantly change under the exposure to P_4_ or E_2_ (Figure 2). The calculated rate of 26 ± 2 nmol/min/mg of cells (control) is comparable with that of HeLa cells (21 nmol/min/mg cells, [10]). The lactate concentration is somewhat lower (∼1 mM) than that observed in HeLa cells at 5 mM glucose (27 mM, [10]). Glucose uptake was unchanged by E_2_ and P_4_ treatment (control: 8.0 ± 1.4; E_2_: 7.6 ± 1.1; P_4_: 9.5 ± 1.6 nmol/min/mg protein). Considering that each glucose molecule gives two lactate molecules, these values suggest that the majority of the glucose use in the cell is for glycolysis under all three conditions.

**Figure 3:**
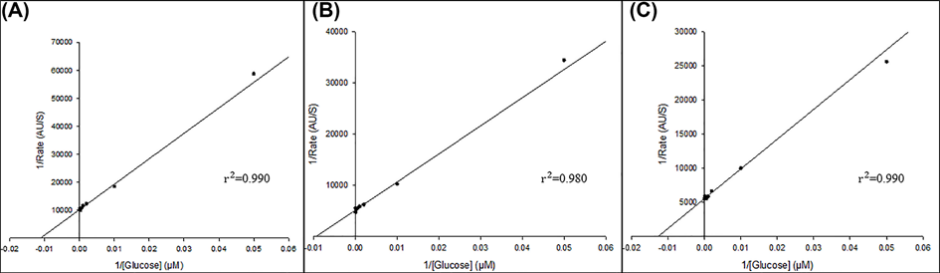
HK Lineweaver–Burk plots Initial rates measured in GMMe cytosolic enzyme extract (*n*=1, 5 mM Glucose) for control (**A**), 10 µM P_4_ (**B**) and 10 nM E_2_ (**C**).

Also, pyruvate and PEP levels were unchanged by hormone treatment (PEP: 5.8 ± 1.6, 5.8 ± 2.2, 8.45 ± 3.3 mM, *n*=5; pyruvate: 6.1 ± 2.0, 7.5 ± 2.9, 11.5 ± 1.6 mM, *n*=5) for control, E_2_ and P_4_, respectively. These concentrations are similar to levels observed in AS-30D rat cells [31]. Because they are more than ten-times higher than the *K*_m_*s* as substrates for PYK and LDH, respectively, those enzymes remain essentially saturated with PEP or pyruvate under all three conditions. However, to determine if, under physiological conditions, the enzymes are at *V*_max_, it would be necessary to determine the concentration of the other substrates: ADP (PYK) and NADH (LDH).

Alterations in PGM kinetics by hormones may affect glycogen metabolism, as E_2_ and P_4_ treatment decrease the *V*_max_ and increase *K*_m_ to G1P relative to control. At cellular G1P concentrations (4.5 mM, unchanged by hormones), PGM activity was reduced by 31% for P_4_ treatment but 89% for E_2_ treatment, suggesting that glycogen metabolism may be strongly inhibited under exposure to E_2_ and, unexpectedly, slightly inhibited by P_4_ treatment. Thus, decreased PGM activity may be part of the mechanism by which E2 enhances insulin’s stimulatory effects on glycogen storage. Such inhibition would not raise glycogen levels in the absence of increased glucose supply.

## Discussion

We have measured for the first time the effect of hormones (E_2_ and P_4_) treatment on both the activities (*V*_max_) and kinetics of the enzymes of glycolysis in a single model system (GMMe). Furthermore, it is the first time for such complete measurements in uterine cells derived from an animal with an obligate diapause phase of reproduction. The results are markedly different from other mammals *in vivo* but consistent with patterns observed previously for *in vitro* studies and GMMe [22,24]. While these hormones alone *in vitro* did not alter glycolytic flux, metabolite levels and the glucose consumption in the GMMe cells, treatment with E_2_ induced a decrease in most enzyme activities, while P_4_ alone induced an increase in several *K*_m_s.

Glycolytic enzyme expression, but not activity, has been measured previously both in live mink and in cultured mink endometrial cells. GMMe cells express GAPDH and HK-1, and GAPDH mRNA levels are not changed by P_4_ and E_2_ treatment [22,23]. We also found GAPDH activity was unaffected by P_4_ and E_2_ treatment. In live mink, HK expression was shown to increase during estrus [20], whereas GMMe HK *activity* was found to be reduced after exposure to E_2_. We found only one isoform of HK to be present in cytosolic enzyme extract.

Although we found that the activity of most of the glycolytic enzyme, including HK, are decreased by E_2_ treatment of GMMe (Figure 1 and Table 1), in rat uteri E_2_ exposure increased the activities of all enzymes except PGM [10]. There are several explanations for the unique effects of E_2_ on GMMe glycolytic enzymes, in addition to transcriptional effects. It is quite likely that P_4_ and E_2_ act in coordination with other hormones while regulating glycolytic enzymes *in vivo*. Insulin is required for stimulation of *in vitro* glycogen synthesis by E_2_ [22]. Thus, insulin, and possibly other hormones, may also be required to attain the stimulatory effects of E_2_ observed for rat glycolysis.

Regulation of glycolytic enzymes in mink may also be distinct from that of other organisms. Several enzymes within glycolysis are regulated by distinct types of post-translational modifications. For instance, phosphorylation fine-tunes the metabolism network and receives signals from upstream hormones. Thus, enzymes in mink uterine tissue and GMMe cells may exhibit a distinct profile of modifications compared with those in tissues. It is also possible that there is variation in post-translational modifications and glycolytic enzyme isoforms between mink and other mammals such as rats.

We also measured for the first time the *K*_m_s of glycolytic enzymes in immortalized endometrial cells. Pyruvate kinase *K*_m_s were nearly identical to those measured in mink liver [24] (*Km*_PEP_ = 0.209 vs. 0.230 mM GMMe; *Km_ADP_* = 0.380 vs. 0.230 mM, liver vs. GMMe, respectively). For enolase, *Km*_2PG_ was lower in mink than in *Rattus norvegicus*, whereas for LDH *Km*_Lac_ was substantially higher in mink (Table 1). For PGM, *Km*_G1P_ increased more than 40-fold with exposure to E_2_ (26 ± 15 to 1200 ± 346 µM) (Table 1). Thus, PGM velocity would be decreased by 89% under these conditions. This suggested that either the expression of a low-affinity PGM isoform is induced by E_2_ or post-translational modification, essentially preventing G1P from being converted into G6P. This is consistent with the fact that E_2_ blocks glycogen mobilization in mink uterine epithelial cells [22]. Because E_2_ is unable to stimulate glycogen synthesis in GMMe cells in the absence of insulin [22], the inhibition of PGM by E_2_ may be permissive rather than sufficient for glycogen synthesis.

If the decreases in glycolytic enzyme activities are priming glucose metabolism for regulation by insulin, alone P_4_ and E_2_ would not necessarily have any effects on the glycolytic flux. We did not observe any significant changes in overall lactate production for GMMe cells (Figure 2). While E_2_ has been shown, in a few studies in rats, to increase uterine glycolytic flux [13,32,33], these studies have been of recently harvested tissue, putatively after exposure to insulin. This is congruent with recent studies that found an important role for insulin in mediating the effects of uterine hormones [22,23]. E_2_ has been previously shown to work in coordination with insulin on uterine carbohydrate metabolism. For instance, E_2_ increases insulin receptors over two-fold in the uteri of ovariectomized rats [34].

Similarly, no significant changes were observed for glucose transport in GMMe. In rat uterine tissues, glucose uptake and consumption were increased by E_2_ exposure *in vivo* [32,33]. P_4_ increased glucose transporter expression in sheep [34] and mice [35] where E_2_ also decreased expression. However, the levels of insulin *in vivo* were not controlled and were presumably non-zero. Thus, E_2_ and P_4_ *in vivo* were likely acting in concert with physiological levels of insulin or insulin-like growth factor (IGF1) to affect transport. There is currently no evidence that E_2_ and P_4_ regulate glucose transport without insulin, as glucose transporters in human cells *in vitro* were not directly regulated by E_2_ or P_4_ [36].

The inability of E_2_ and P_4_ treatment to affect glucose usage in GMMe, despite substantial changes in enzyme activities, supports the growing evidence that these ovarian hormones are not able to act independently to affect uterine metabolism and development [22,23]. Studies performed before the identification of IGF1 asserted that uterine glycogen synthesis was independent of insulin [35]. This dogma needs reassessment. Hormones such as insulin and IGF1 appear to be needed to couple reproduction to energy metabolism across a range of organisms and their tissues [36,37]. If this mechanism extends to humans, the insulin resistance that underlies metabolic syndrome and associated female infertility (reviewed [38]) may be connected to the inability of insulin to affect the ability of ovarian hormones (E_2_ and P_4_) to properly change carbohydrate use in uterine tissues.

Finally, although kinetic analysis and flux measurement provide insight into GMMe glycolysis, there is not a simple relationship between changes in enzyme kinetics, fluxand the behavior of a system. Recent studies have demonstrated that the kinetics and kinetic mechanisms of enzymes measured in physiological conditions can be used to create effective models of metabolism [39,40]. Thus, our data could be used to construct a computational model for GMMe similar to that of HeLa [29,30], which demonstrated an effective approach to integrating *in vitro* data to construct a quantitative model of nutrient metabolism using COPASI software [41]. With such a model, we could develop hypotheses for future experiments. For instance, we could predict the effects of changes in blood glucose or determine how hormones affect which enzymes exert the most control over the carbohydrate allocation.

## Supplementary Material

Supplementary Figures S1-S2

## Abbreviations

ADP: adenosine diphosphate
ALDO: aldolase (E.C 4.1.2.13)
ATP: adenosine triphosphate
BCA: bicinchoninic acid
DHAP: dihydroxyacetone phosphate
DMSO: dimethyl sulfoxide
ENO: enolase (E.C. 4.2.1.11)
E_2_: 17-β-estradiol
F1,6BP: fructose 1,6-bisphosphate
F6P: fructose 6-phosphate
GAPDH: glyceraldehyde-3-phosphate dehydrogenase
G1P: glucose 1-phosphate
G3P: glyceraldehyde 3-phosphate
G6P: glucose 6-phosphate
G6PDH: glucose-6-phosphate dehydrogenase (E.C. 1.1.1.49)
HK: hexokinase (E.C. 2.7.1.1)
HPI: phosphohexose isomerase (E.C. 5.3.1.9)
IGF1: insulin-like growth factor
LDH: lactate dehydrogenase (E.C. 1.1.1.27)
NAD(H): nicotinamide adenine dinucleotide
PEP: phosphoenolpyruvate
PFK-1: phosphofructokinase type I (E.C. 2.7.1.11)
PGaM: phosphoglycerate mutase (E.C. 2.7.2.3)
PGK: phosphoglycerate kinase (E.C. 5.4.2.1)
PGM: phosphoglucomutase (E.C. 5.4.2.2)
PYK: pyruvate kinase (E.C. 2.7.1.40)
P_4_: progesterone
TIM: triosephosphate isomerase (E.C. 5.3.1.1)
2PG: 2-phosphoglycerate
3PG: 3-phosphoglycerate
