# Supplementary material for: Estradiol and progesterone affect enzymes but not glucose consumption in a mink uterine cell line (GMMe)
Source: Biosci Rep. 2020 Apr 24;40(4):BSR20193512. doi: 10.1042/BSR20193512 (PMC7182661; doi:10.1042/BSR20193512)
Supplement: Supplementary Figures S1-S2 [file BSR-2019-3512_supp.pdf]

Supplemental Figure 1

A. Hexokinase

i) *Glucose varied*

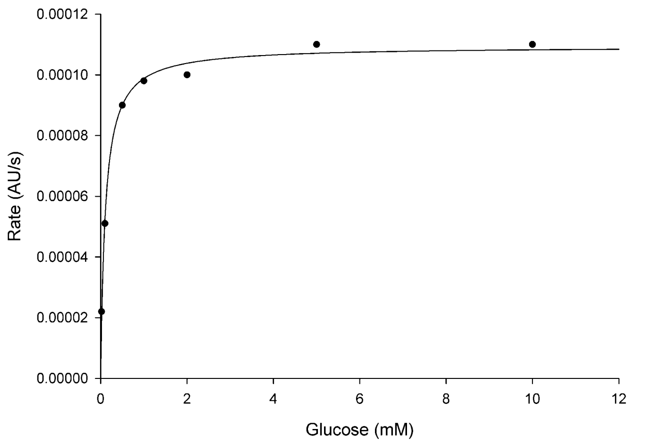

Km= 0.108 mM

ii) *ATP Varied*

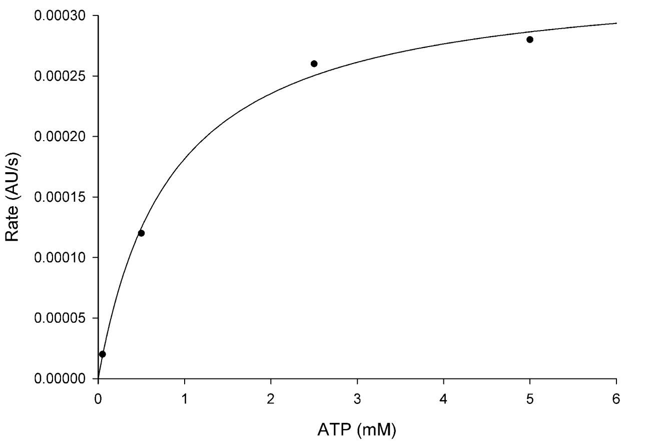

Km=0.843 mM

B. Glucose-6-Phosphate Dehydrogenase

i) *G6P varied*

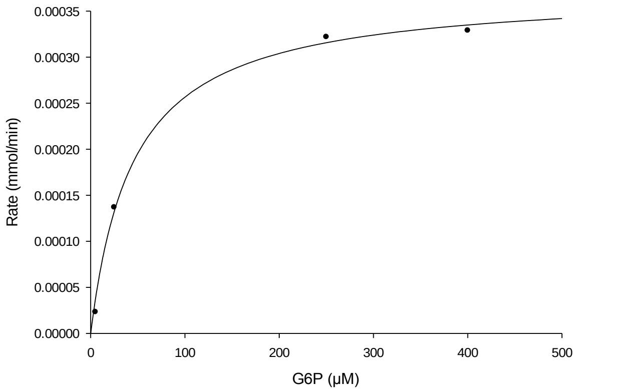

Km=45.2 μM

ii) *$NADP^+$ varied*

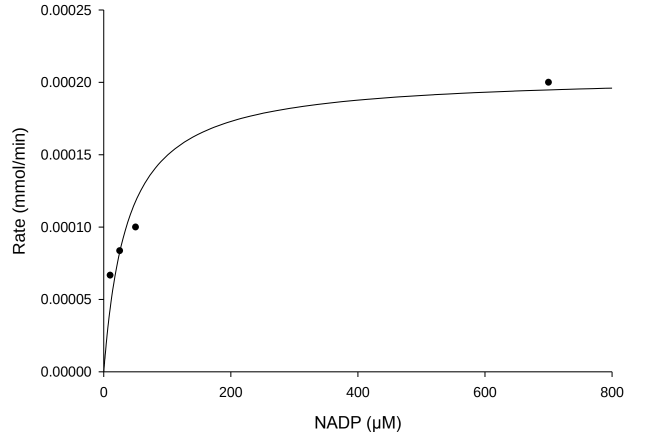

Km=37.1 μM

C. Phosphoglucomutase

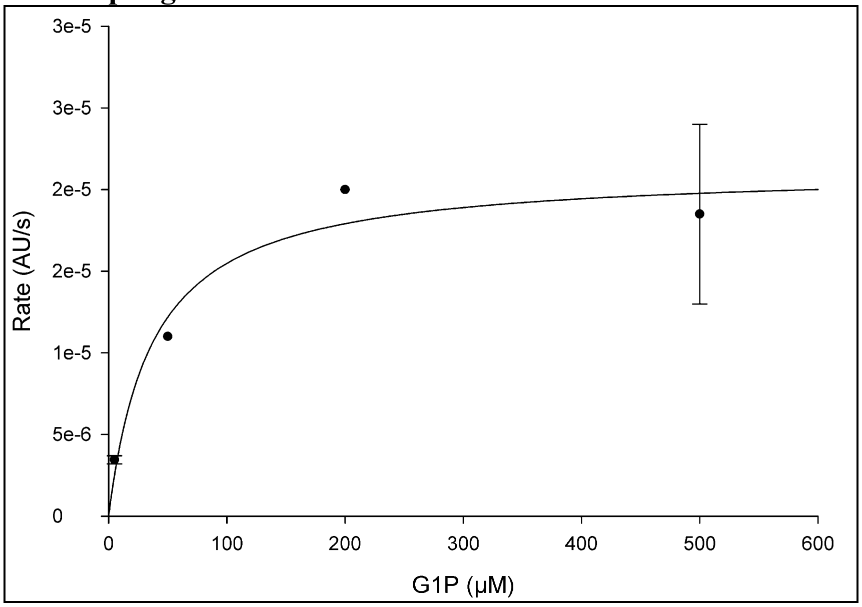

Km= 37.2 μM

D. HPIr:

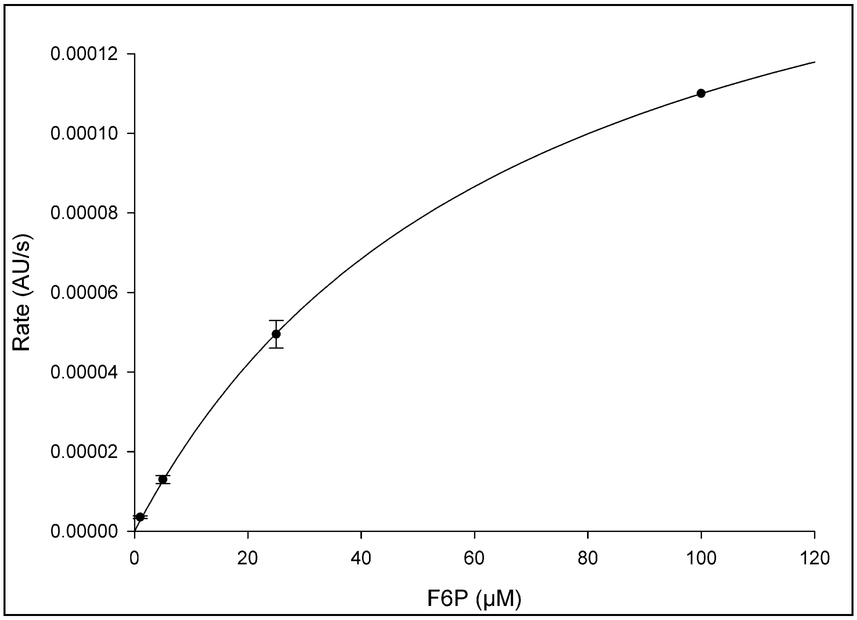

Km = 67.8 μM

E. Aldolase

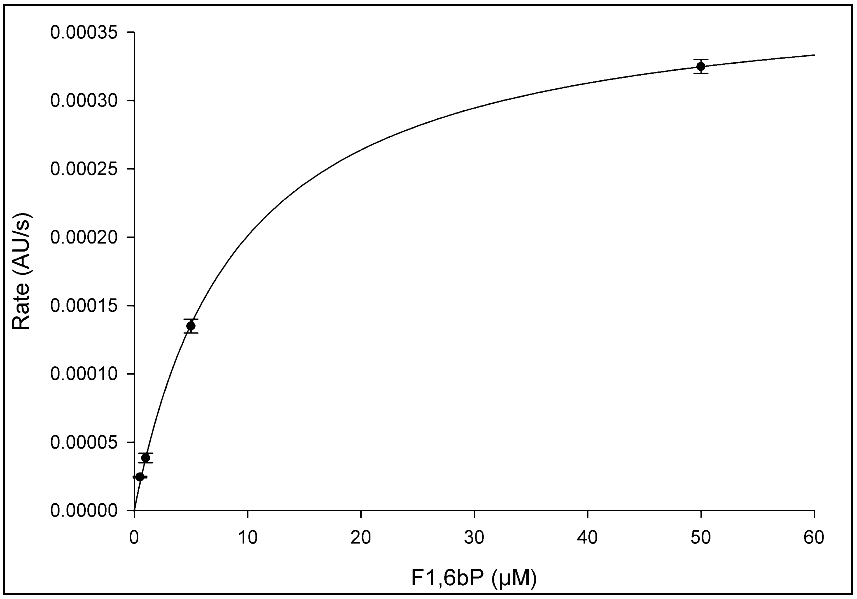

Km=9.1 μM

F. TIM*f*

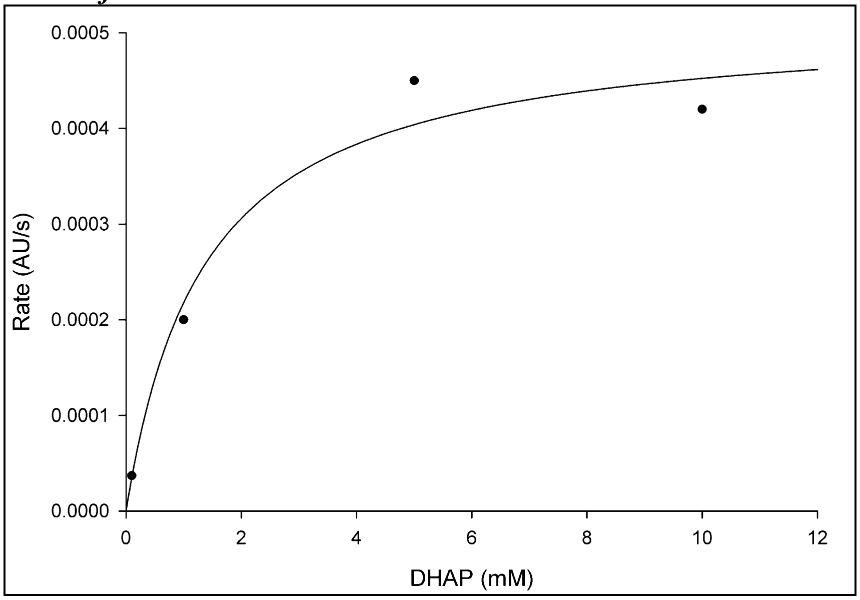

Km =1.4 mM

G. TIM*r*

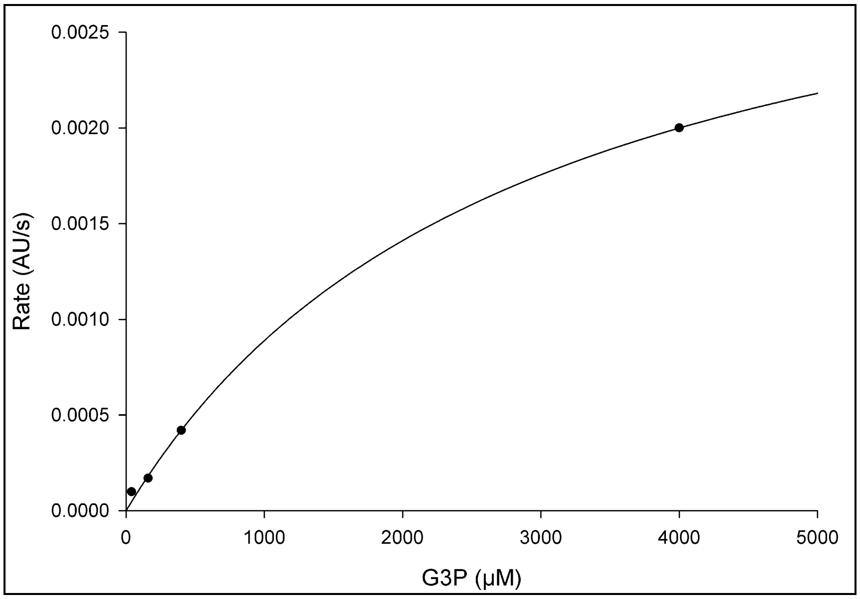

Km=2853 µM

H. PGK*r*

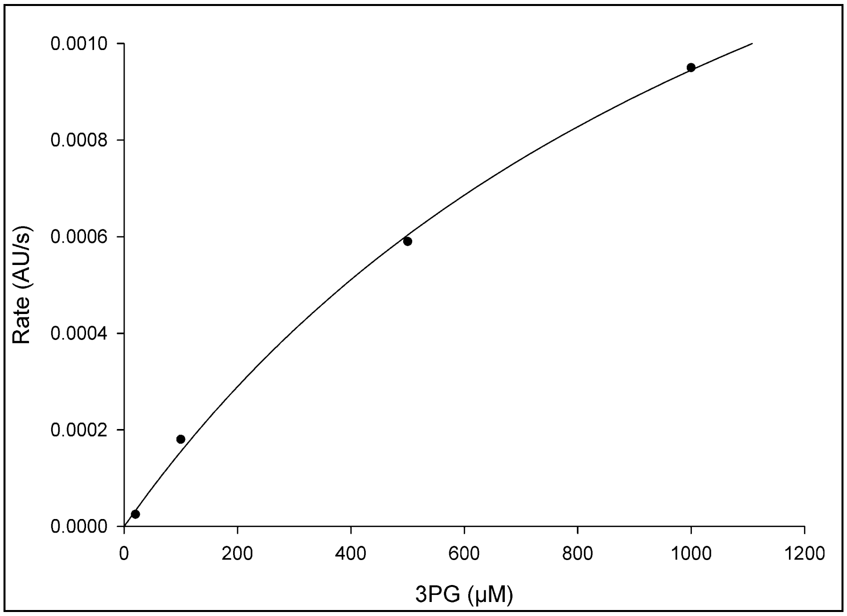

Km=1580 µM

I. PhosphoglycerateMutase (PGaM)

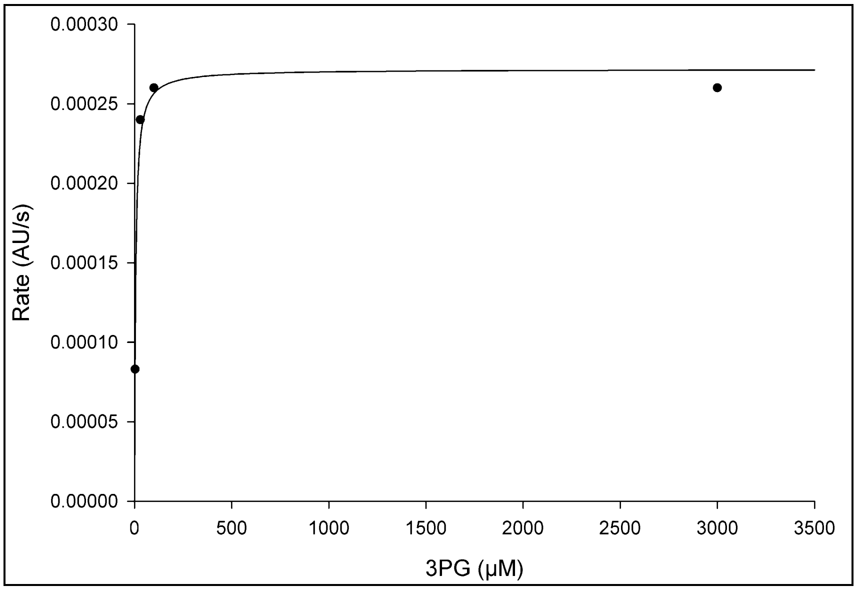

Km= 5.9 µM

J. Enolase

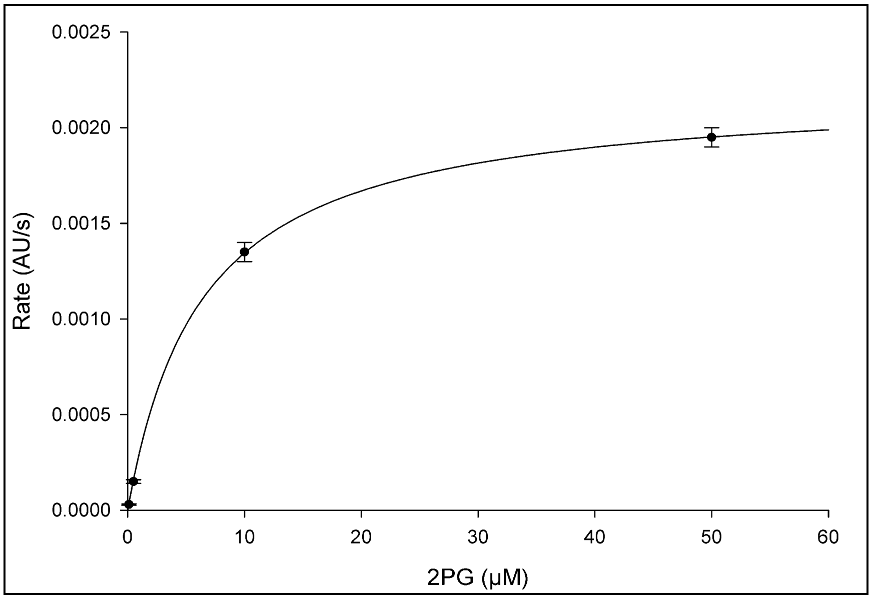

Km= 6.3 µM

K. Pyruvate Kinase

i) *PEP varied*

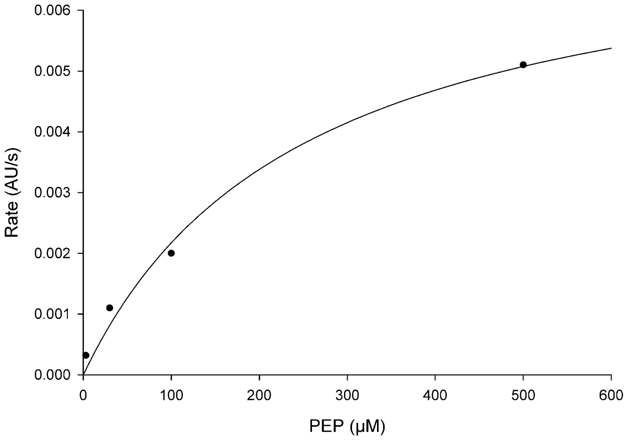

Km= 251 μM

ii) *ADP varied*

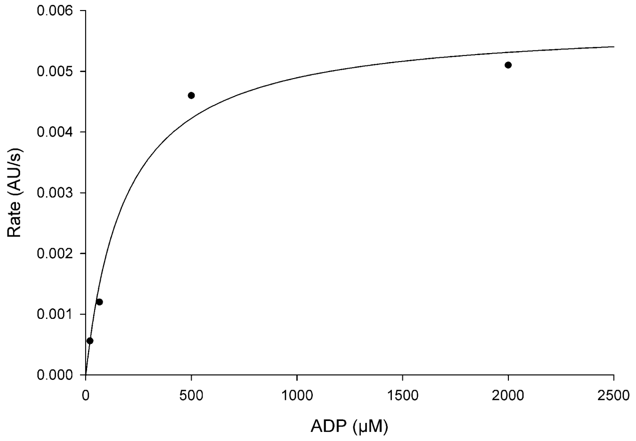

Km= 187 μM

L. Lactate Dehydrogenase (forward)

i) *Pyruvate varied*

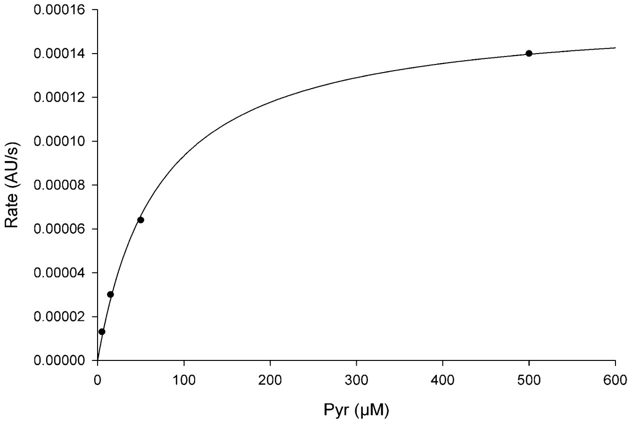

Km=71 μM

ii) *NADH varied*

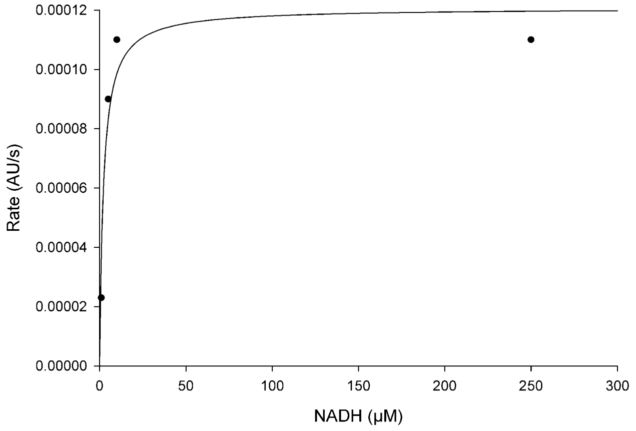

Km=2.2 μM

M. Lactate Dehydrogenase (reverse)

i)*Lactate varied*	ii) *NAD*$^{+}$ *varied*
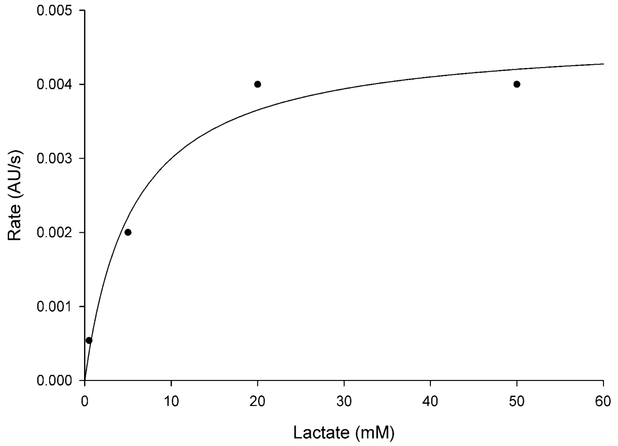	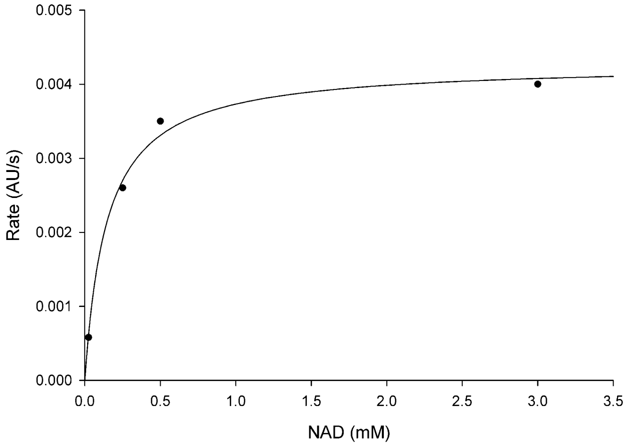
Km=5.6 mM	Km=146 μM

Supplemental Figure 1: Representative Michaelis-Menten curve fits for each enzyme with single measurements for each point unless otherwise noted: A. Hexokinase (HK), i) glucose varied at 10 mM ATP and ii) ATP varied at 3 mM Glucose; B. Glucose-6-Phosphate dehydrogenase (G6PDH), i) G6P varied at 700 μM $NADP^+$ and ii) $NADP^+$ varied at 400 μM G6P; C. Phosphoglucomutase (PGM); D. Hexose Phosphate isomerase (reverse) (HPIr); E. Aldolase (forward) mean ±SD in duplicate; F. Triose phosphate isomerase (TIM*f*) G. Triose phosphate isomerase reverse (TIM*r*); H. Phosphoglycerate kinase reverse (PGK*r*); I.Phosphoglycerate Mutase (PGaM);J. Enolase, mean± SD in duplicate; K. Pyruvate Kinase (PYK) i. PEP varied at 1 mM ADP ii. ADP varied at 0.6 mM PEP; L. Lactate dehydrogenase forward (LDH*f*) i. pyruvate varied at 150 μM NADH ii. NADH varied at 300 μM pyruvate M. Lactate dehydrogenase reverse (LDH*r*) i) lactate varied at mM NAD^+ ii) NAD+ varied at mM lactate;

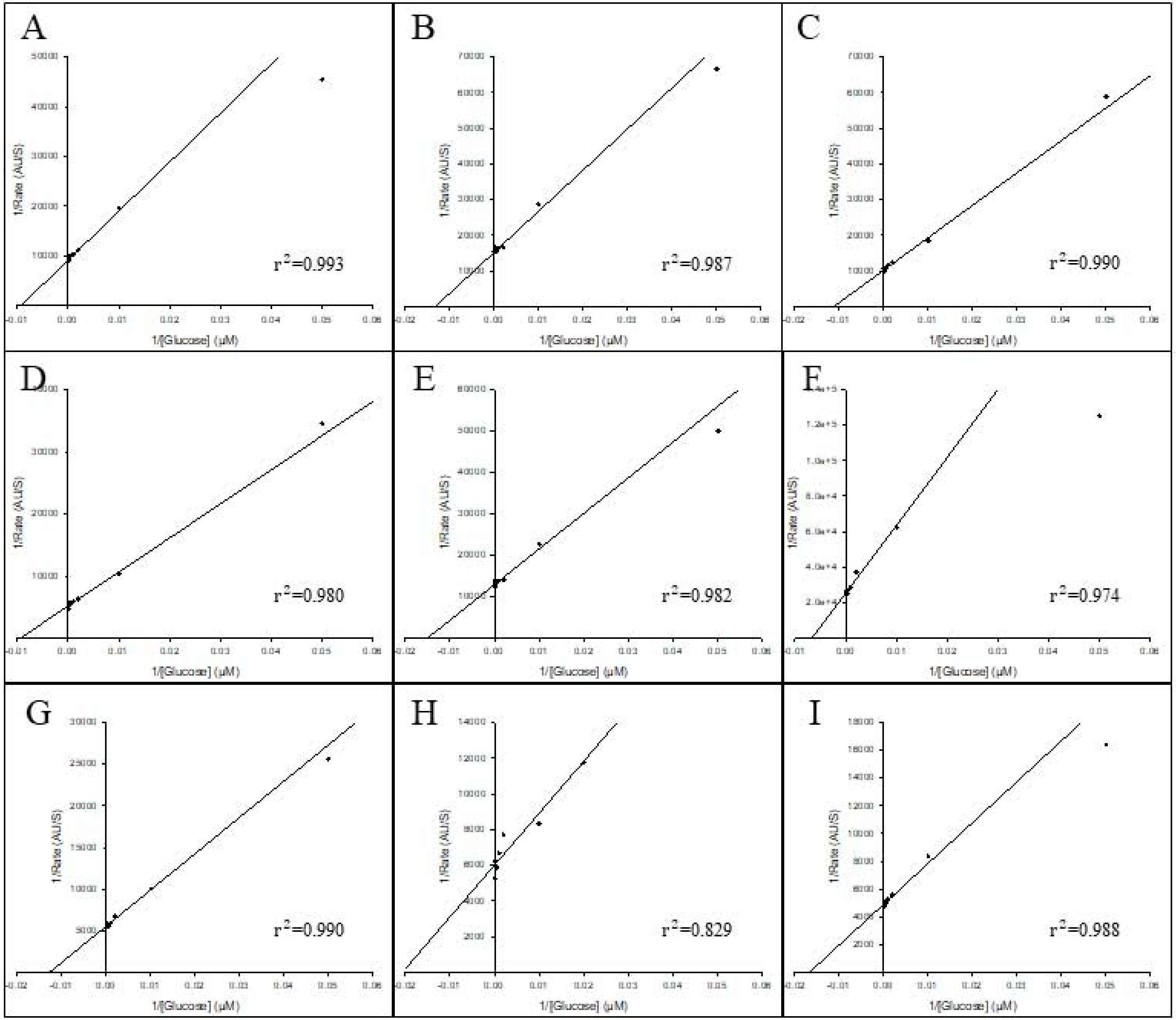

A
r^2=0.993
B
r^2=0.987
C
r^2=0.990
D
r^2=0.980
E
r^2=0.982
F
r^2=0.974
G
r^2=0.990
H
r^2=0.829
I
r^2=0.988
1/Rate (AU/S)
1/[Glucose] (μM)

Supplemental Figure 2: HK Lineweaver-Burk plots. Three independent assays for control (A-C), 10 μM P_4 (D-F), and 10 nM E_2 (G-I). Initial rates measured in GMMe cytosolic enzyme extract (n=1, 5 mM Glucose).
